# Expanding the treatment envelope for brain therapy: simulation models and head phantoms

**DOI:** 10.1186/2050-5736-3-S1-O31

**Published:** 2015-06-30

**Authors:** Matt Eames, Gianmarco Pinton, Mohamad Khaled, W Jeff Elias, John Snell, Laurent Marsac, Arik Hananel, Mickael Tanter, Wilson Miller, Neal Kassell, Max Wintermark, Jean-Francois Aubry

**Affiliations:** 1 Focused Ultrasound Foundation, Charlottesville, VA, United States; 2 Institut Langevin, Paris, France; 3 University of Virginia, Charlottesville, VA, United States

## Background/introduction

Thermal therapy is currently limited to central areas of the brain in order to maximize the antenna gain between the outer cortex and the target. So far, clinical applications have been limited to thalamotomies for neuropathic pain, essential tremor and Parkinsonian tremor. We developed numerical simulations and head phantoms in order to investigate the possibility to target more eccentric targets in the brain in silico and *in vitro*.

## Methods

### Simulations

The acoustic field was modeled with a 3D finite difference fullwave acoustic code developed at Institut Langevin. CT measurements of human heads were converted to acoustical maps. Two types of hemispheric therapeutic transducers were modeled: a low frequency 220 kHz transducer, and a 1 MHz transducer that required aberration correction with a time reversal approach. The therapeutic pulses propagated across multiple skull lengths to establish a stable reverberation pattern.

### Head phantoms

Three different models of phantoms have been developed: cadavers, gel-filled skulls, and a head mold containing a skull and filled with gel that mimics the brain and the skin. Each was positioned in a clinical tcMRgFUS system, sonicated at 1100 acoustic watts for 12 seconds at different locations under MR thermometry monitoring.

## Results and conclusions

Simulations: For an equivalent energy deposition rate, it is shown that at high frequency the volume of the brain that is above a set of MI thresholds is over 3 orders of magnitude smaller than for the low frequency, and it is entirely confined to a compact focal spot. High frequency transducers seem more suited to expand the treatment envelop towards the skull base and the low frequency to expand the treatment envelop towards the skull vault and to target larger volumes. Head phantoms: The cadaveric phantom, gel-filled skull, and full head models had heating efficiencies of 5.3, 4.0, and 3.9 °C/kW/s, respectively, compared to a heating efficiency of 2.6 °C/kW/s for a sample of essential tremor clinical treatments.

